# Supplementary material for: The interactome of intact mitochondria by cross-linking mass spectrometry provides evidence for coexisting respiratory supercomplexes
Source: Mol Cell Proteomics. 2017 Dec 8;17(2):216–32. doi: 10.1074/mcp.RA117.000470 (PMC5795388; doi:10.1074/mcp.RA117.000470)
Supplement: Supplemental Data [file supp_RA117.000470_133922_0_supp_23978_fzffwf.zip › spectra_annotation/mito_spectra_annotation/423-1-19-1-8-1.pdf]

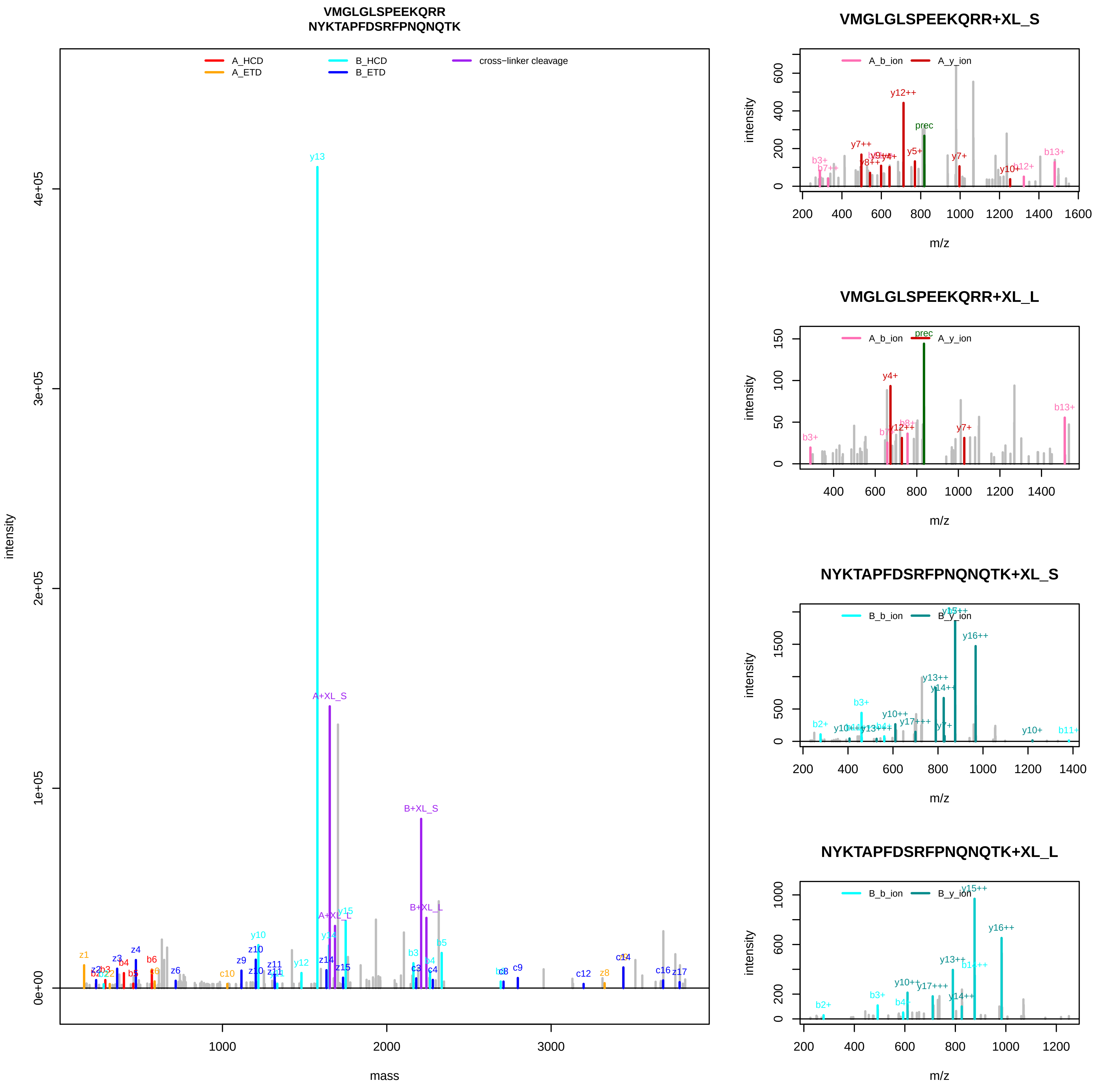

VMGLGLSPEEKQRR
NYKTAPFDSRFPNQNQTK
A_HCD
A_ETD
B_HCD
B_ETD
cross−linker cleavage
intensity
mass
0e+00
1e+05
2e+05
3e+05
4e+05
1000
2000
3000
y13
A+XL_S
B+XL_S
A+XL_L
B+XL_L
y15
y14
y10
y12
z1
z2
z3
z4
z6
z9
z10
z11
z14
z15
b3
b4
b5
b6
c3
c4
c8
c9
c10
c12
c14
c16
z8
z17
VMGLGLSPEEKQRR+XL_S
VMGLGLSPEEKQRR+XL_L
NYKTAPFDSRFPNQNQTK+XL_S
NYKTAPFDSRFPNQNQTK+XL_L
A_b_ion
A_y_ion
B_b_ion
B_y_ion
m/z
prec
y12++
y7++
y5+
y7+
y10+
y4+
b3+
b13+
b12+
y15++
y16++
y13++
y14++
y10++
y17+++
b2+
b11+
